# Implications of the HIV testing protocol for refusal bias in seroprevalence surveys

**DOI:** 10.1186/1471-2458-9-163

**Published:** 2009-05-28

**Authors:** Georges Reniers, Tekebash Araya, Yemane Berhane, Gail Davey, Eduard J Sanders

**Affiliations:** 1 Institute of Behavioral Science (Population Program), University of Colorado, Boulder CO, USA; 2 School of Social Sciences and School of Public Health, University of the Witwatersrand, Johannesburg, South Africa; 3 Office of Population Research, Princeton University, Princeton NJ, USA; 4 School of Public Health, Addis Ababa University, Addis Ababa, Ethiopia; 5 Centralized School of Nursing, Addis Ababa University, Addis Ababa, Ethiopia; 6 Addis Continental Institute of Public Health, Addis Ababa, Ethiopia; 7 Kenya Medical Research Institute, Centre for Geographic Medicine Research, Kilifi, Kenya; 8 Nuffield Department of Clinical Medicine, Centre for Tropical Medicine, University of Oxford, Oxford, UK

## Abstract

**Background:**

HIV serosurveys have become important sources of HIV prevalence estimates, but these estimates may be biased because of refusals and other forms of non-response. We investigate the effect of the post-test counseling study protocol on bias due to the refusal to be tested.

**Methods:**

Data come from a nine-month prospective study of hospital admissions in Addis Ababa during which patients were approached for an HIV test. Patients had the choice between three consent levels: testing and post-test counseling (including the return of HIV test results), testing without post-test counseling, and total refusal. For all patients, information was collected on basic sociodemographic background characteristics as well as admission diagnosis. The three consent levels are used to mimic refusal bias in serosurveys with different post-test counseling study protocols. We first investigate the covariates of consent for testing. Second, we quantify refusal bias in HIV prevalence estimates using Heckman regression models that account for sample selection.

**Results:**

Refusal to be tested positively correlates with admission diagnosis (and thus HIV status), but the magnitude of refusal bias in HIV prevalence surveys depends on the study protocol. Bias is larger when post-test counseling and the return of HIV test results is a prerequisite of study participation (compared to a protocol where test results are not returned to study participants, or, where there is an explicit provision for respondents to forego post-test counseling). We also find that consent for testing increased following the introduction of antiretroviral therapy in Ethiopia. Other covariates of refusal are age (non-linear effect), gender (higher refusal rates in men), marital status (lowest refusal rates in singles), educational status (refusal rate increases with educational attainment), and counselor.

**Conclusion:**

The protocol for post-test counseling and the return of HIV test results to study participants is an important consideration in HIV prevalence surveys that wish to minimize refusal bias. The availability of ART is likely to reduce refusal rates.

## Background

Progress in medical technology has brought rapid HIV testing within reach of nationally representative surveys. This has generated new prospects for resolving bias in HIV prevalence estimates based on antenatal clinic (ANC) sentinel surveillance data, or, for providing a new gold standard for HIV prevalence estimates altogether [1-5]. However, data from population-based surveys are also subject to bias due to the exclusion of high risk groups from the sampling frame, and non-response because of population mobility and refusal. The association between mobility and HIV infection has been documented extensively [6-9]. In comparison, relatively little is known about the relationship between refusal and HIV infection in nationally representative surveys [2,3,5,10]. Several small-scale studies in STD and antenatal clinics have concluded that refusals are positively associated with HIV infection [11-19]. Three studies remain inconclusive about the nature of the relationship or suggest the opposite pattern [20-22]. On aggregate, population-based surveys are believed to underestimate true HIV prevalence, but most studies have not been able to identify significant bias due to testing refusal [5,9,23-27]. Two studies challenge that optimism [28,29].

A study design feature that may contribute to differences in refusal bias is the protocol for post-test counseling and the return of test results. Agreeing to post-test counseling and the return of test results is often a prerequisite of study participation in health facility-based studies. This contrasts with most population-based surveys that follow a protocol in which respondents or clients do not receive their HIV test results (e.g., Demographic and Health Surveys). Instead, they are given a voucher for retesting at the nearest Voluntary Counseling and Testing (VCT) center at no cost (if the service is not already free of charge) [30]. In early studies involving testing for HIV, the return of test results was not usually an option because samples had to be shipped to an off-site lab for analysis. With the increasing availability and reliability of rapid tests, the return of test results is now feasible in the same session in which the specimens are collected. Because of the ethical prescription that study participants should share in the benefits of research [30], the pressure to provide post-test counseling including the return of HIV test results in HIV prevalence surveys is likely to increase in the future. Therefore, it is important to assess how to accommodate this guideline in the testing protocol while preserving or maximizing the external validity of the ensuing HIV prevalence estimates.

Using data from a health facility in Addis Ababa, we first present covariates of testing refusal. Second, we quantify refusal bias in HIV prevalence estimates under different post-test counseling study protocols via regression models that account for sample selection. A final noteworthy feature of our study is that antiretroviral therapy (ART) was introduced in Ethiopia during the course of data collection, and that allows us to evaluate its impact on refusal rates.

## Methods

The data for this study come from prospective monitoring of hospital admissions and outpatient visits which was initiated at Zewditu Memorial Hospital in May 2003 and continued for nine months. Zewditu Memorial Hospital is a government facility in the inner city of Addis Ababa and was one of the few hospitals with a VCT center of sufficient capacity to accommodate our study. Initially, the study covered the TB-HIV clinic (TB, ambulatory patients), the medical emergency (ER), internal medicine (IM), gynaecology (GY), and pediatric wards (PE). For each patient, a ward nurse collected basic background characteristics (age and sex) as well as the admission and discharge diagnosis. One month into the study, the surgical ward (SU) was included and we added educational status, religion, birthplace and marital status as background variables on the data collection forms. After new patients were identified, a VCT-nurse did pre-test counseling and asked for written consent of the patient. For minors, consent was obtained from the parent or guardian along with the assent of the patient him- or herself.

Following pre-test counseling, patients had the option to participate in the study with the return of HIV test results and post-test counseling (consent level A), to participate in the study without the return of test results or post-test counseling (consent level B), or, to decline testing and counseling altogether (consent level C). In the remainder of this article, we refer to post-test counseling as the interaction between counselor and client that includes the return of the test result and a counseling session tailored to the HIV status of the client. The three consent levels help us mimic refusal bias under different post-test counseling study protocols: we consider consent level C to represent refusals in a protocol where post-test counseling is not offered to respondents (or where an explicit provision exists to test without post-test counseling), and we combine consent levels B and C to represent refusals in a protocol where post-test counseling is a requirement of study participation.

After consent was obtained, the VCT-nurse administered a Determine Rapid HIV1-2 test. Capillus™ HIV-1/HIV-2 confirmatory tests were done on positive samples, and if the outcomes of these tests were discrepant a Uni-Gold™ HIV test was done as a tie breaker. Tests were offered free of charge. Nine VCT nurses carried out counseling and anywhere between two and four nurses covered each ward. All but one of the counselors were female.

To study whether refusals are more common among patients with a higher likelihood of infection, we rely on the admission diagnosis because (1) it is correlated with HIV status, and (2) also observed for patients who were not tested. We use admission rather than discharge diagnosis because it is less likely to be influenced by the test result itself. The availability of information on the medical condition of respondents constitutes an important advantage of a medical facility-based sample. In contrast, most measured traits correlate weakly with HIV status in community-based studies, and that renders assessments of refusal bias in HIV prevalence estimates questionable. The downside of a medical facility-based study is that it is not necessarily representative of the determinants of participation in population-based surveys (e.g., levels of -undisclosed- prior knowledge of one's HIV status may be higher in a health facility sample, and prior knowledge of HIV positive status has been identified as a source of bias in HIV prevalence estimates [29]). Therefore, our estimates of the degree of refusal bias cannot be extrapolated to general population surveys, but a health facility-based sample is probably satisfying to identify the type of post-test counseling protocol that minimizes bias (i.e., to identify the relative magnitude of refusal bias under different study protocols).

All admission diagnoses were coded using the International Classification of Diseases (ICD-10) [31]. Coders did not have access to HIV status information. For each entry in Table 1, we calculated the HIV prevalence among those who agreed to test. These percentages measure the likelihood of infection, and are used as a predictor of consent. We thus assume that within each group of conditions listed in Table 1, HIV status is not correlated with the willingness to be tested (e.g., that the HIV prevalence in patients with pneumonia is the same for those who accepted and those who refused the test). We use the likelihood of infection variable rather than dummies for the admission diagnoses for simplicity. Substituting one for the other does not change the substantive conclusions from this study. The pseudo R^2 ^in a logistic regression of HIV status on the likelihood of infection is 0.25.

**Table 1:** Admission diagnoses and likelihood of infection. Zewditu Memorial Hospital, Addis Ababa (2003–04, age 16 and above)

	% HIV+	N	ICD-10 code
Diarrhoea and GE of presumed infectious origin	66.7	42	A09
Respiratory TB	69.7	33	A15–16
Other TB	60.0	15	A17–19
HIV	100.0	2	B2
Malaria	17.1	35	B50–54
Herpes zoster, oral candiasis, toxoplasmosis and PCP	94.7	38	B02, B37, B58–59
Other infectious and parasitic diseases	14.0	43	A01, A03, A07, A30, A35, A41, A63–64, A68, A75, A82, B45
Neoplasm's of breast, cervix, uterus and leiomyoma	14.6	48	C50, C53–55, D25–26
Other neoplasms (benign and malignant)	0.0	25	C0, C2–4, C51–52, C56–58, C6–9, D0, D22–24, D3–4
Thyroid disorders	9.9	71	E00–05
Diabetes and hypoglycemia	11.1	27	E10–E16
Diseases of the nervous system (mainly meningitis)	35.7	14	G00, G03–04, G25, G40, G54
Hypertension	7.1	28	I10–I13
Hypotension	61.9	21	I95
Other diseases of the circulatory system	6.7	45	I05, I09, I15, I21, I31, I38, I49–51, I61, I63–64, I80, I83–I84, I86, I88
Pneumonia	30.6	36	J18
Other diseases of the respiratory system	26.9	26	J11, J44–46, J86, J90, J93–94, J98
Gastritis and other diseases of the oesophagus, stomach and duodenum	15.0	60	K27, K29–31
Diseases of the appendix	9.0	78	K35, K37–38
Hernia and intestinal obstruction	5.7	70	K40, K42–43, K46, K56
Cholelithiasis and diseases of the pancreas	6.1	132	K80, K82, K85–K86
Other diseases of the digestive system	15.8	38	K04, K12, K60, K62–63, K65–66, K72–73, K75–76, K83, K91–93
Diseases of the skin and subcutaneous tissue	22.2	9	L, M
Glomerular diseases and diseases of the urinary system	13.6	22	N0–3
Diseases of male genital organs	2.6	38	N4
Inflammatory diseases of female pelvic organs and disorders of the female genital tract	20.0	25	N7–9
Complications of pregnancy and delivery	15.9	44	O
Fever of unknown origin	32.7	104	R50
Chronic illness	79.3	29	R69
Symptoms signs and abnormal clinical findings not elsewhere specified	17.5	63	R0–4, R56–58, R62
External causes and injuries	7.7	39	S, T, X
Other and unknown admission diagnoses	12.9	31	A80, B19, B56, D5–8, E15, E40–42, E55, E83, E86, E88, K36, P07, Q43, Q53, U, Z4

Total	22.2	1331	

The likelihood of infection as measured by the admission diagnosis is first used as a predictor in logistic regression models with the consent level as the outcome. In these models, we verify whether the effect of the infection likelihood persists while controlling for other characteristics of the respondent. In the next step, we estimate refusal bias in HIV prevalence via a comparison of observed HIV prevalence estimates and predicted values generated by Heckman probit models that account for sample selection [32-34]. The Heckman sample selection model corrects for the possibility that HIV prevalence is different in respondents who refuse testing. More formally, the Heckman sample selection model is a two-equation model consisting of a regression and a selection equation that are simultaneously estimated. The regression equation predicts HIV status: *y = Xβ + u*_1 _where X is a vector of covariates. The selection equation specifies that HIV status is only observed if Zγ + *u*_2 _> 0. In this equation Z stands for a vector of characteristics that affect consent for HIV testing. The error terms in both equations are assumed to be normally distributed. Ordinary probit estimates of the parameters in the regression equation are biased when *ρ *(the correlation between *u*_1 _and *u*_2_) is not zero. The Heckman selection model lets us use information for patients who refused the HIV test (e.g., counselor, admission diagnosis, and other sociodemographic background characteristics) to improve estimates of parameters in the regression model, and thus improve estimates of HIV prevalence (i.e., the mean predicted value).

We limit the study population in four respects. The first set of excluded cases is multiple admissions of the same individual. We only consider first admissions because higher order admission diagnoses might be influenced by the test outcome at first visit, and thus introduce problems of reverse causality. For the same reason, we exclude individuals who volunteered their HIV status. The third excluded category is patients under 16 years old, primarily because we wish to restrict our study population to an age range that is common in seroprevalence surveys. The TB/HIV clinic constitutes another special case. HIV testing is standard practice in diagnosing patients of the TB/HIV clinic and some are referred to it precisely for that reason. The TB/HIV clinic of Zewditu Memorial Hospital was also one of the pioneering ART facilities in Ethiopia, which contributes to the (self-) selection of patients.

### Ethics

The study protocol was approved by the Research and Publications Committee of the Addis Ababa University Faculty of Medicine and received ethics clearance from the Ethiopian Science and Technology Agency, and the Institutional Review Board of the University of Pennsylvania. Written informed consent was obtained for administering and using the HIV test results for research purposes. No individual informed consent was requested for using (anonimized) background characteristics and the admission diagnosis of patients who refused the HIV test.

## Results

### Study descriptives

In total 2719 individuals were approached. After excluding the TB/HIV clinic patients and those under 16 years, 1650 cases were retained (Table 2). Fifty-four of them were discharged prior to testing and 49 already knew their HIV status. These cases are omitted from further analysis. Of all patients approached, 86.1% consented to testing (consent levels A and B), and 75.5% chose testing followed by a post-test counseling session (consent level A). The percentage of total refusals (13.9%, consent level C) is of the same magnitude as those observed in the DHS involving serostatus testing in Mali, Kenya and Zambia [5]. Of those in consent levels A and B, 29.7% tested positive. The share of positives is markedly higher among those who declined post-test counseling (consent level B, 49.6%) compared to those who agreed to testing and post-test counseling (consent level A, 27.5%).

**Table 2:** Consent for testing and HIV status (Zewditu Memorial Hospital, Addis Ababa, 2003–04)

	Freq.	Column %	Study participants (column %)	HIV prevalence
Consent level A (testing & post-test counseling)	1168	70.8	75.5	27.5
Consent level B (testing only)	164	9.9	10.6	49.6
Consent level C (total refusal)	215	13.0	13.9	unknown
Known HIV status	49	3.0	excluded	81.8
Discharged/expired prior to testing	54	3.3	excluded	
				
Total	1650	100		

### Covariates of consent

Table 3 shows associations between patients' background characteristics and consent. A three-category variable for religion (Orthodox Christian, Muslim and other), and a dichotomous variable for place of birth (Addis Ababa versus elsewhere) are weak and statistically insignificant predictors of consent and therefore not shown. The age effects are suggestive of an inverse U-shaped pattern with refusals peaking in middle-aged adults. Refusal rates also co-vary by marital and educational status. The most pronounced variability in consent is, however, not by patient characteristics, but by ward and counselor. The first is possibly related to the reason for admission (and thus HIV status), but could be confounded by the variable success of counselors in enrolling study participants. Several counselors have refusal rates (consent level C) below 10%. For others, the refusal rate varies between 20 and 43%. Refusals also declined following the introduction of ART.

**Table 3:** Covariates of consent for HIV testing (Zewditu Memorial Hospital, Addis Ababa, 2003–04)

	**Consent level (row %)**					**Consent level (row %)**			
**Age^a^**	A	B	C	Total	**Counselor^b^**	A	B	C	Total
16–19	86.0	4.7	9.4	107	1	57.5	13.7	28.8	73
20–29	73.9	11.7	14.5	498	2	91.4	5.0	3.6	303
30–39	68.7	14.1	17.2	396	3	53.6	3.6	42.9	28
40–49	77.3	9.7	13.0	247	5	73.2	26.8	0.0	41
50–59	77.5	10.6	12.0	142	6	0.0	0.0	100.0	2
60+	85.9	3.9	10.3	156	7	98.5	0.3	1.2	322
Pearson Chi^2^(10) = 28.99 p < .01					8	63.1	16.6	20.3	728
Missing	100.0	0.0	0.0	1	9	56.0	10.0	34.0	50
					Pearson Chi^2^(14) = 276.27, p < .01				
**Education**									
Illiterate	82.3	10.2	7.5	362	**Ward**				
1–6^th ^grade	76.5	11.8	11.8	272	ER	76.2	13.9	9.9	625
7–12th grade	76.0	11.0	13.0	607	GY	55.2	9.5	35.3	201
>12^th ^grade	61.2	10.9	27.9	129	IM	57.7	20.6	21.7	97
Pearson Chi^2^(8) = 37.46 p < .01					SU	84.1	6.1	9.8	624
Missing	68.9	7.9	23.2	177	Pearson Chi^2^(6) = 134.44, p < .01				
									
**Marital status**					**Study month^a^**				
Single	78.6	11.6	9.8	481	Prior to ART	64.0	16.2	19.8	445
Mar	76.5	9.0	14.6	769	Since ART	80.1	8.4	11.5	1,102
Div/wid	69.5	17.8	12.7	118	Pearson Chi^2^(2) = 44.72, p < .01				
Pearson Chi^2^(4) = 14.49, p < .01									
Missing	67.0	10.1	22.9	179	**Gender**				
					Female	75.3	9.9	14.7	876
**Likelihood of infection^a^**					Male	75.7	11.5	12.8	671
≤ 7.49	83.5	6.4	10.1	376	Pearson chi^2^(2) = 1.86, p = 0.40				
7.5 – 14.9	79.0	8.6	12.5	409					
15.0 – 29.9	69.4	10.6	20.0	376					
≥ 30	69.9	16.9	13.3	385					
Pearson Chi^2^(6) = 44.05, p < .01									
Missing	0.0	100.0	0.0	1					

Notes:

^a ^In the regression models in Tables 4 and 5, age is defined in terms of single year age groups and study month is coded 0 for the period prior to the introduction of ART and consecutive numbers for months that followed. HIV likelihood is used as the proportion HIV+ for each ICD-10 entry in Table 1. The other variables are defined as shown in the table.

^b ^Counselor #4 only worked in the TB/HIV clinic and omitted from this table and any subsequent analysis. Counselor 6 worked primarily in the pediatrics ward.

Particularly relevant for the analysis of bias in HIV-prevalence estimates is the association between the likelihood of infection and refusal: consent for testing and post-test counseling (consent level A) drops from over 83% in patients with the lowest likelihood of infection to just under 70% among those with the highest likelihood of infection. This is partly compensated by an increasing share of patients who consented to testing without post-test counseling (consent level B) as the likelihood of infection increased.

To explore the relationship between refusal and its predictors in a multivariate context, we use logistic regression models with the consent level as the outcome of interest (Table 4). In the first binary logistic model (consent levels B and C versus A), the likelihood of being HIV positive is correlated with consent for testing and post-test counseling and highly significant: for each percentage point increase in the likelihood of infection, the odds to consent to testing and post-test counseling (consent level A) decrease by 1.5%. The analysis also confirms that counselors had variable success in obtaining consent. Of further interest is that refusals gradually declined following the introduction of ART. In Model 2, we introduce a number of additional control variables (i.e., ward of admission, male gender, age, educational level and marital status). The odds to consent to testing and post-test counseling are twice as high for women as for men. The quadratic effect of age corroborates the curvilinear relationship between age and consent described in Table 3. Those with higher educational status are less likely to participate in testing, which also supports the bivariate results. In terms of marital status, singles are most likely to consent to testing and post-test counseling. The parameter estimates for the infection likelihood, counselor, and study month, however, hardly change in the presence of these controls.

**Table 4:** Binary and multinomial logistic regressions predicting refusal of testing for HIV (Zewditu Memorial Hospital, Addis Ababa, 2003–04)

	Binary logistic regression predicting refusal (exp(b) or odds ratios)		Multinomial logistic regression predicting refusal (exp(b) or relative risk ratios)			
	B & C versus A		B versus A	C versus A	B versus A	C versus A
	Model 1	Model 2	Model 3		Model 4	
Likelihood of infection	1.01**	1.01**	1.02**	1.01**	1.01**	1.01**
						
Counselor (vs #1)						
Counselor 2	0.07**	0.07**	0.12**	0.05**	0.06**	0.09**
Counselor 3	0.46	-	0.09**	0.71	-	-
Counselor 5	0.50	0.33	1.58	-	1.48	-
Counselor 6	-	-	-	-	-	-
Counselor 7	0.01**	0.01**	0.01**	0.02**	0.00**	0.01**
Counselor 8	0.46**	0.44	0.56	0.42**	0.26	0.68
Counselor 9	0.65	0.56	0.43	0.78	0.38	0.76
						
Study month (vs period prior to ART)	0.82**	0.81**	0.78**	0.84**	0.73**	0.88*
						
Ward (vs ER)						
GY		1.23			0.44	2.68**
IM		1.42			0.56	2.73**
SU		0.83			0.60	1.04
						
Male		1.96**			1.68**	2.30**
						
Age		1.05			1.13**	1.02
Age squared		.999*			.998**	1.00
						
Education (vs no schooling)						
Grade 1–6		1.33			1.16	1.57
Grade 7–12		1.27			0.79	1.95**
> 12^th ^grade		1.70*			0.82	2.85**
						
Marital status (vs never married)						
Married		1.44*			1.15	1.71**
Sep/Div/Wid		1.92*			1.78	2.03
						
N	1544	1357"	1546		1359	
LR chi^2 ^(df)	354.57(8)	364.68(18)	406.49(18)		453.04(38)	
Prob > chi^2^	0.00	0.00	0.00		0.00	
Pseudo R^2^	0.21	0.25	0.18		0.24	
Log likelihood	-680.93	-554.13	-917.05		-732.04	

Notes:

* p ≤ .10; ** p ≤ .05

See Table 3 and the notes to that table for a definition of the explanatory variables. Other variables that were controlled for, but omitted in the final models because they lack statistical significance are: birth region (Addis Ababa versus other); religion (Orthodox Christian versus other); a squared term for likelihood of infection; an interaction between the likelihood of infection and study month; an interaction between birth region and sex. Because education and marital status were only introduced as additional variables in the second month of the study, models two and four are based on fewer cases.

Breaking down the outcome by level of consent (models 3 and 4) changes little in terms of the substantive conclusions compared to the binary logistic regression models. The most noteworthy differences are that age is a weak predictor of total refusal (consent level C versus A), and that educational status does not have an effect in the equation predicting testing without versus testing with post-test counseling (consent level B versus A). The parameters for marital status point in the same direction as in the binomial model but vary in their significance level.

### Bias in HIV prevalence estimates

To quantify refusal bias in HIV prevalence estimates, we turn to Heckman sample selection models of HIV prevalence. We use a Heckman probit model to generate predicted values of HIV prevalence, and compare these with estimates from standard probit models. All explanatory variables in models 2 and 4 of Table 4 are used in the selection equation of the Heckman model. The Heckman regression equation predicting HIV status includes age, a squared term for age, sex, the likelihood of being HIV positive, and marital status. These variables are of little substantive interest in this study, and are simply chosen to maximize the predictive power of the regression equation. Table 5 presents HIV prevalence estimates based on standard probit models and Heckman probit models under different scenarios. The bottom row shows the likelihood ratio (LR) test for the hypothesis that the error terms in the regression and selection equations are uncorrelated (*H*_0_: *ρ *= 0), or, in other words, that selection bias in negligible.

**Table 5:** Comparison of HIV seroprevalence estimates based on standard probit models and models accounting for sample selection under various scenarios (Zewditu Memorial Hospital, Addis Ababa, 2003–04)

	Scenario		
	Test of Heckman model^a^	Post-test counseling is required	Post-test counseling is optional
E(HIV% – Probit)	17.7 (16.4 – 19.1)	17.8 (16.6–19.1)	21.4 (20.2–22.7)
E(HIV% – Heckman)	23.1 (21.7 – 24.4)	23.4 (22.1–24.7)	23.7 (22.4–25.0)
Observed HIV%	22.2 (19.9 – 24.4)	unknown	unknown
Sample	Consent groups A and B	All consent groups	All consent groups
Assumption	HIV status in consent group B is unobserved	HIV status in consent groups B and C is unobserved	HIV status in consent group C is unobserved
LR test H_0_:ρ = 0	p < .01	p < .01	p = .07

Notes: 95%- CI are reported between brackets. Using dummies for admission diagnosis rather than the likelihood of infection in these regressions hardly changes the estimated prevalence rates though one of the selection models did not converge.

^a ^In the first column, we assume that HIV status in consent group B is unknown, and compare the ordinary Probit and Heckman selection model estimate with the true or observed value of HIV prevalence.

In the first column we present a simple empirical test of the Heckman model. Here we assume that we do *not *have information on HIV status for those who agreed to test but declined post-test counseling (consent level B), and we predict HIV prevalence in the sample consisting of individuals in consent levels A and B only using an ordinary probit model and a probit model that accounts for sample selection. Because we know the HIV prevalence in the total sample, we can compare the probit estimates with observed HIV prevalence. The ordinary probit estimate of HIV prevalence is 17.7%, the selection model establishes HIV prevalence at 23.1%, and the true or observed value is 22.2%. Heckman estimates are thus more accurate than standard probit estimates of HIV prevalence. The LR test confirms that selection bias is significant.

It is noteworthy that *ρ *= 0 for a Heckman model that only includes basic sociodemographic background characteristics (sex, age, marital status, and education) in the selection equation (not shown). That model also underestimates HIV prevalence. Adding counselor to the selection equation renders *ρ *≠ 0, but significantly overestimates HIV prevalence. Inclusion of information on the health status of patients – an indicator that correlates well with HIV status and consent for testing – thus considerably improves Heckman predictions of HIV prevalence. This sensitivity analysis confirms an earlier finding that the validity of Heckman estimates are subject to the specification of the selection equation [34]. The specification used in this application, however, produces good estimates of HIV prevalence.

The last two columns compare HIV estimates for two plausible study protocols. Bias in prevalence estimates is substantial if response is dichotomized into refusal or full participation without the option of testing without post-test counseling (column 2). This scenario is most typical for clinical intervention studies. Bias is much smaller, and only marginally statistically significant, when the study protocol explicitly allows participants to opt out of post-test counseling and the return of HIV test results. This is shown in the third column. This scenario is more typical for population-based serosurveys.

## Discussion

Our analyses establish that consent for testing is correlated with the likelihood of HIV infection (assessed in terms of the diagnosis at admission): patients who agree to testing with or without post-test counseling (consent levels A and B) are less likely to be infected than those who refuse an HIV test (consent level C). This relationship implies that testing refusal constitutes a potential source of bias in HIV prevalence estimates. Regression methods that account for sample selection confirm this, but qualification is required in two respects. First, our study is based on a hospital population and demands confirmation in a more general sample. Second, much seems to depend on the study protocol and informed consent procedures. In this sample, bias is limited if respondents are offered the opportunity to opt out of post-test counseling and the return of test results.

Because most population-based surveys utilize a testing protocol that does not necessarily involve post-test-counseling and the return of test results, they are less likely to be affected by refusal bias than studies where post-test counseling is a requirement for study participation. This does not mean, however, that HIV prevalence estimates from population-based serosurveys are free of bias. First, we identified marginally significant bias under the assumptions of a protocol whereby test results are not returned to respondents. Second, bias may result from other sources than those studied here (e.g., limitations of the sampling frame and other forms of non-response).

Although this paper has focused on the relationship between the likelihood of infection and consent for testing, it is not the most important predictor of consent. The largest variation in consent is produced by the counselors, which suggests that studies interested in minimizing non-response must be careful in the selection and training of their fieldwork team. Unfortunately our study was not designed to assess the possible reasons for the variable study enrollment rates by counselor (e.g., via the randomization of counselors across wards). We have no reason, however, to suspect significant bias in HIV prevalence estimates due to variability in consent attributable to counselors. Another covariate of consent is the availability of ART. In our study, the odds to consent for testing and counseling increased by about 20% per month following the launch of a governmental ART program. The absence of a control group, however, does not allow us to exclude other factors that may be responsible for this association. The finding that patients are more likely to agree to testing once treatment becomes available is nonetheless plausible, and confirms findings from another observational study [35].

## Conclusion

The protocol for post-test counseling and the return of HIV test results to study participants is an important determinant of consent for testing, and should be carefully evaluated in studies that wish to minimize refusal bias in HIV prevalence surveys. For the sake of scientific accuracy, it is recommended to provide a modality to test without post-test counseling when introducing the study protocol to respondents. In studies where there is a long wait between testing and the availability of test results, this is often a de-facto option. As technological advances in rapid testing methods reduce the waiting time, however, this will become a consideration of increasing importance. To date, most population-based serosurveys have followed a protocol that did not involve the return of HIV test results. To the extent that our findings can be extrapolated to non health facility-based settings, this study suggests that in doing so, these surveys have avoided a potentially important source of bias. Finally, we find that the availability of ART is likely to reduce refusal rates, and thus the potential for refusal bias.

## Competing interests

The authors declare that they have no competing interests.

## Authors' contributions

The data collection protocol was developed by TA, ES, and YB. TA supervised fieldwork. GR conceived the study and carried out the analysis. TA, GD, YB and ES contributed to the study design. All authors contributed to the interpretation of the data, drafting the manuscript, and approved its final version.

## Pre-publication history

The pre-publication history for this paper can be accessed here:


